# Supplementary material for: GECOBench: a gender-controlled text dataset and benchmark for quantifying biases in explanations
Source: Front Artif Intell. 2026 Jan 5;8:1694388. doi: 10.3389/frai.2025.1694388 (PMC12813014; doi:10.3389/frai.2025.1694388)
Supplement: Supplementary file 1 [file Data_Sheet_1.pdf]

Supplementary Material for GECOBench

Supplementary Material for GECO and GECOBench

1 GECO DATASHEET

1.1 Motivation

For what purpose was the dataset created? Was there a specific task in mind? Was there a specific gap that needed to be filled? Please provide a description.

In light of the wide adoption of complex machine learning systems based on deep neural networks and their deployment in critical decision-making contexts, researchers and developers have sought techniques to provide answers about their inner workings. Due to their opacity, deep neural networks are particularly perceived as 'black-box' models, prompting the development of Explainable Artificial Intelligence (XAI) methods. However, such methods operate on opaque premises, leading to a lack of clear statements expressing the formal problem of XAI. This development has led to a branch of research focused on quantitatively validating XAI methods.

Large pre-trained language models have become an important backbone of many downstream tasks in natural language processing (NLP). However, these models are trained on a large collection of datasets containing a variety of biases, including gender biases. Currently, it is unclear to what extent such biases impact model explanations in unfavorable ways. The dataset at hand, GECO, provides a gender-controlled text corpus in which otherwise identical sentences appear in male and female forms. As a result, we obtain ground truth 'world explanations' for gender classification tasks with labels 'male' vs. 'female', facilitating the objective evaluation of XAI methods and their future development.

Who created this dataset (e.g., which team, research group) and on behalf of which entity (e.g., company, institution, organization)?

This dataset has been constructed by the research group "QAI Labs" at the Technische Universität Berlin Universität[1].

Who funded the creation of the dataset? If there is an associated grant, please provide the name of the grantor and the grant name and number.

This result is part of a project that has received funding from the European Research Council (ERC) under the European Union's Horizon 2020 research and innovation programme (Grant agreement No. 758985), the German Federal Ministry for Economy and Climate Action (BMWK) in the frame of the QI-Digital Initiative.

[1] https://qai-labs.org/

1.2 Composition

What do the instances that comprise the dataset represent (e.g., documents, photos, people, countries)? Are there multiple types of instances (e.g., movies, users, and ratings; people and interactions between them; nodes and edges)? Please provide a description.

This dataset contains otherwise identical sentences in both a male and female version. Ground-truth explanations come in the form of binary sequences where each entry corresponds to a word in the sentence, indicating if the word is a discriminative feature.

How many instances are there in total (of each type, if appropriate)?

GECO consists of two datasets, D_S and D_A, constructed from the same original sentence scraped from Wikipedia. In dataset $\mathcal{D}_S$, we altered only the human subject, whereas in dataset $\mathcal{D}_A$, all words referring to humans were modified. Both datasets consist of a training and test dataset encompassing 2576 and 644 sentences, respectively.

Does the dataset contain all possible instances or is it a sample (not necessarily random) of instances from a larger set? If the dataset is a sample, then what is the larger set? Is the sample representative of the larger set (e.g., geographic coverage)? If so, please describe how this representativeness was validated/verified. If it is not representative of the larger set, please describe why not (e.g., to cover a more diverse range of instances, because instances were withheld or unavailable).

No.

What data does each instance consist of? "Raw" data (e.g., unprocessed text or images) or features? In either case, please provide a description.

Each instance in the dataset consists of a sentence represented as a list of words, accompanied by a binary sequence of equal length indicating the discriminative words for gender classification, used as ground truth explanations. Additionally, each sample includes the index of the corresponding unaltered sentence in its original form.

Is there a label or target associated with each instance? If so, please provide a description.

Each sample includes a target class, either 0 or 1, denoting whether the human entities in the original sentence scraped from Wikipedia were converted to females or males, respectively. For D_S, only the subject was modified, whereas for D_A, all human entities were altered.

Is any information missing from individual instances? If so, please provide a description, explaining why this information is missing (e.g., because it was unavailable). This does not include intentionally removed information, but might include, e.g., redacted text.

No.

Are relationships between individual instances made explicit (e.g., users' movie ratings, social network links)? If so, please describe how these relationships are made explicit.

Each Wikipedia-sourced sentence was transformed into both a male and a female version, establishing a relationship between the modified and otherwise identical sentences. The 'sentence_idx' field enables matching each sample with the corresponding sample of the opposite gender.

Are there recommended data splits (e.g., training, development/validation, testing)? If so, please provide a description of these splits, explaining the rationale behind them.

We already provide a standard data split: training/validation and holdout (testing) set.

Are there any errors, sources of noise, or redundancies in the dataset? If so, please provide a description.

GECO consists of sentences altered by the authors of this work, which naturally suffer from human-made labeling errors, even though we applied great efforts to minimize such effects.

Is the dataset self-contained, or does it link to or otherwise rely on external resources (e.g., websites, tweets, other datasets)? If it links to or relies on external resources, a) are there guarantees that they will exist, and remain constant, over time; b) are there official archival versions of the complete dataset (i.e., including the external resources as they existed at the time the dataset was created); c) are there any restrictions (e.g., licenses, fees) associated with any of the external resources that might apply to a future user? Please provide descriptions of all external resources and any restrictions associated with them, as well as links or other access points, as appropriate.

GECO was created using sentences sourced from Wikipedia, but the resulting datasets themself are self-contained and do not rely on external resources.

Does the dataset contain data that might be considered confidential (e.g., data that is protected by legal privilege or by doctor-patient confidentiality, data that includes the content of individuals non-public communications)? If so, please provide a description.

No, the sentences were sourced from publicly accessible Wikipedia articles on books that are part of Project Gutenberg's top 100 book list[2].

Does the dataset contain data that, if viewed directly, might be offensive, insulting, threatening, or might otherwise cause anxiety? If so, please describe why.

As sentences are scraped from Wikipedia articles describing book plots, they may contain offensive, insulting, or threatening topics derived from the content of the books.

Does the dataset relate to people? If not, you may skip the remaining questions in this section.

No.

Does the dataset identify any subpopulations (e.g., by age, gender)? If so, please describe how these subpopulations are identified and provide a description of their respective distributions within the dataset.

[2] `https://www.gutenberg.org/browse/scores/top`

[N/A]

Is it possible to identify individuals (i.e., one or more natural persons), either directly or indirectly (i.e., in combination with other data) from the dataset? If so, please describe how.

[N/A]

Does the dataset contain data that might be considered sensitive in any way (e.g., data that reveals racial or ethnic origins, sexual orientations, religious beliefs, political opinions or union memberships, or locations; financial or health data; biometric or genetic data; forms of government identification, such as social security numbers; criminal history)? If so, please provide a description.

[N/A]

1.3 Collection Process

How was the data associated with each instance acquired? Was the data directly observable (e.g., raw text, movie ratings), reported by subjects (e.g., survey responses), or indirectly inferred/derived from other data (e.g., part-of-speech tags, model-based guesses for age or language)? If data was reported by subjects or indirectly inferred/derived from other data, was the data validated/verified? If so, please describe how.

The dataset is constructed from raw sentences extracted from Wikipedia articles summarizing plots of the top 100 books listed on Project Gutenberg, following a series of preprocessing steps outlined below to prepare the data for gender classification. We then utilize these preprocessed sentences to derive the final GECO datasets by manually annotating words corresponding to human entities and creating male and female versions of each sentence. For dataset D_S, we modify only the human subject, whereas for dataset $\mathcal{D}_A$, all human entities are modified.

During the manual labelling process, we ensured the quality and suitability of the sentences scraped automatically from Wikipedia.

What mechanisms or procedures were used to collect the data (e.g., hardware apparatus or sensor, manual human curation, software program, software API)? How were these mechanisms or procedures validated?

We used web scraping using Selenium[3] to scrape the top 100 book list from Project Gutenberg on the 17th of March 2022, followed by an automated Google search to retrieve the corresponding Wikipedia articles for each title. Subsequently, we scraped the webpage content and used a combination of natural language processing (NLP) preprocessing via spaCy[4] and manual preprocessing steps listed below to filter the raw sentences. The manual annotation process, which involved labeling human entities, was performed utilizing an Excel spreadsheet generated specifically for this purpose, including the original filtered sentences alongside placeholders for modified sentences featuring annotated human entities.

If the dataset is a sample from a larger set, what was the sampling strategy (e.g., deterministic, probabilistic with specific sampling probabilities)?

No.

Who was involved in the data collection process (e.g., students, crowdworkers, contractors) and how were they compensated (e.g., how much were crowdworkers paid)?

The raw sentences were sourced automatically using web-scraping techniques. The annotations were collected from our lab members, including student and research assistants. All members were compensated according to the standardized wage agreement.

Over what timeframe was the data collected? Does this timeframe match the creation timeframe of the data associated with the instances (e.g., recent crawl of old news articles)? If not, please describe the timeframe in which the data associated with the instances was created.

[3] https://www.selenium.dev/

[4] https://spacy.io/

The raw sentences used in GECO were scraped on the 17th of March 2022. Since the list of the top 100 books on Project Gutenberg consists of classical books and novels, we expect the wikipedia articles to be mature.

Were any ethical review processes conducted (e.g., by an institutional review board)?If so, please provide a description of these review processes, including the outcomes, as well as a link or other access point to any supporting documentation.

No.

Does the dataset relate to people?If not, you may skip the remaining questions in this section.

No.

Did you collect the data from the individuals in question directly, or obtain it via third parties or other sources (e.g., websites)?

[N/A]

Were the individuals in question notified about the data collection?If so, please describe (or show with screenshots or other information) how notice was provided, and provide a link or other access point to, or otherwise reproduce, the exact language of the notification itself.

[N/A]

Did the individuals in question consent to the collection and use of their data?If so, please describe (or show with screenshots or other information) how consent was requested and provided, and provide a link or other access point to, or otherwise reproduce, the exact language to which the individuals consented.

[N/A]

If consent was obtained, were the consenting individuals provided with a mechanism to revoke their consent in the future or for certain uses?If so, please provide a description, as well as a link or other access point to the mechanism (if appropriate).

[N/A]

Has an analysis of the potential impact of the dataset and its use on data subjects (e.g., a data protection impact analysis) been conducted?If so, please provide a description of this analysis, including the outcomes, as well as a link or other access point to any supporting documentation.

[N/A]

1.4 Preprocessing/cleaning/labeling

Was any preprocessing/cleaning/labeling of the data done (e.g., discretization or bucketing, tokenization, part-of-speech tagging, SIFT feature extraction, removal of instances, processing of missing values)?If so, please provide a description. If not, you may skip the remainder of the questions in this section.

Yes. The raw dataset consists of all sentences extracted from Wikipedia pages, which are unlabeled and noisy. To ensure the quality of this dataset and prepare it for use in a gender classification task, we employed a series of pre-processing steps to clean the data.

To refine the raw data, we implemented a set of criteria, eliminating sentences exceeding 30 tokens in length, those with neutral subjects (typically denoted by the word 'it'), and those lacking punctuation, author mentions, or duplicate information. Additionally, we excluded sentences without common nouns related to humans, subjects not integral to the plot, and those containing citations or proper nouns appearing only once, as these elements may not significantly impact the narrative. We ensure that the subject of a sentence either corresponds to proper nouns, pronouns 'he' and 'she', or common nouns referring to a human being. Furthermore, we ensured grammatical consistency within sentences and content relevance, excluding extraneous information about authors or book interpretations.

Was the "raw" data saved in addition to the preprocessed/cleaned/labeled data (e.g., to support unanticipated future uses)?If so, please provide a link or other access point to the "raw" data.

Yes. The original data, including the GECO dataset as well as all related artifacts, can be accessed via the "Open Science Framework" (OSF[5]).

The raw data includes the list of books, the raw text scraped from Wikipedia, all extracted sentences and the excel sheet used to annotate the data.

Is the software used to preprocess/clean/label the instances available?If so, please provide a link or other access point.

Yes. The pre-processing of our dataset can be found in the Git Repository available at `https://github.com/braindatalab/gecobench`, including instructions on how to run the code for the scraping and pre-processing.

[5] `https://osf.io/74j9s/?view_only=8f80e68d2bba42258da325fa47b9010f`

1.5 Uses

Has the dataset been used for any tasks already? If so, please provide a description.

GECO has not been used for other tasks yet.

Is there a repository that links to any or all papers or systems that use the dataset? If so, please provide a link or other access point.

Does not apply currently.

What (other) tasks could the dataset be used for?

The data we provide is dedicated to analyzing and developing XAI methods applied to pre-trained language models that are fine-tuned on GECO. Technically, this dataset could be employed to perform benchmarks for model fine-tuning in the NLP domain and analyses with respect to gender biases.

Is there anything about the composition of the dataset or the way it was collected and preprocessed/cleaned/labeled that might impact future uses? For example, is there anything that a future user might need to know to avoid uses that could result in unfair treatment of individuals or groups (e.g., stereotyping, quality of service issues) or other undesirable harms (e.g., financial harms, legal risks) If so, please provide a description. Is there anything a future user could do to mitigate these undesirable harms?

GECO is an unbiased dataset regarding grammatical gender by construction, yet we cannot fully rule out the prevalence of other biases, for example, against particular social groups.

Are there tasks for which the dataset should not be used? If so, please provide a description.

GECO, with its two datasets $\mathcal{D}_S$ and $\mathcal{D}_A$, represents a gender classification task purely for the purpose of developing and testing novel XAI methods. And the gender classification task was purely constructed to pursue academic objectives.

1.6 Distribution

Will the dataset be distributed to third parties outside of the entity (e.g., company, institution, organization) on behalf of which the dataset was created?If so, please provide a description.

Yes. We aim to make our dataset publically available under a Creative Commons license hosted on "Open Science Framework" (OSF[6]).

How will the dataset will be distributed (e.g., tarball on website, API, GitHub) Does the dataset have a digital object identifier (DOI)?

We host the dataset via OSF, including a DOI.

When will the dataset be distributed?

The dataset is available on OSF[7].

Will the dataset be distributed under a copyright or other intellectual property (IP) license, and/or under applicable terms of use (ToU)?If so, please describe this license and/or ToU, and provide a link or other access point to, or otherwise reproduce, any relevant licensing terms or ToU, as well as any fees associated with these restrictions.

We will publish the GECO dataset under a Creative Commons license.

Have any third parties imposed IP-based or other restrictions on the data associated with the instances?If so, please describe these restrictions, and provide a link or other access point to, or otherwise reproduce, any relevant licensing terms, as well as any fees associated with these restrictions.

No.

Do any export controls or other regulatory restrictions apply to the dataset or to individual instances?If so, please describe these restrictions, and provide a link or other access point to, or otherwise reproduce, any supporting documentation.

No.

[6] `https://osf.io`

[7] `https://osf.io/74j9s/?view_only=8f80e68d2bba42258da325fa47b9010f`

1.7 Maintenance

Who will be supporting/hosting/maintaining the dataset?

The authors and the QAI Labs research group support the dataset, which is hosted on OSF.

How can the owner/curator/manager of the dataset be contacted (e.g., email address)?

The authors of this dataset can be reached at the e-mail address: `haufe@tu-berlin.de`.

Is there an erratum?If so, please provide a link or other access point.

If errors are found, an erratum will be added to the OSF website containing all meta-information about this dataset.

Will the dataset be updated (e.g., to correct labeling errors, add new instances, delete instances)?If so, please describe how often, by whom, and how updates will be communicated to users (e.g., mailing list, GitHub)?

As we plan to host the dataset as part of an XAI benchmark suite at QAI Labs, updates and error corrections will be part of the maintenance of this benchmark platform; a changelog can be provided via the corresponding website.

If the dataset relates to people, are there applicable limits on the retention of the data associated with the instances (e.g., were individuals in question told that their data would be retained for a fixed period of time and then deleted)?If so, please describe these limits and explain how they will be enforced.

[N/A]

Will older versions of the dataset continue to be supported/hosted/maintained?If so, please describe how. If not, please describe how its obsolescence will be communicated to users.

Older versions of this dataset are hosted on OSF.

If others want to extend/augment/build on/contribute to the dataset, is there a mechanism for them to do so?If so, please provide a description. Will these contributions be validated/verified? If so, please describe how. If not, why not? Is there a process for communicating/distributing these contributions to other users? If so, please provide a description.

We aim to publish this dataset under a Creative Commons license, which will allow other researchers to access it and create derivative work freely.
