# Supplementary material for: GECOBench: a gender-controlled text dataset and benchmark for quantifying biases in explanations
Source: Front Artif Intell. 2026 Jan 5;8:1694388. doi: 10.3389/frai.2025.1694388 (PMC12813014; doi:10.3389/frai.2025.1694388)
Supplement: Supplementary file 2 [file Supplementary_file_1.pdf]

frontiers

Supplementary Material for GECOBench

1 GECO DATASET

1.1 Data Licensing

The titles from Project Gutenberg are available under a public domain license, allowing them to be freely accessed and used by the public. However, our datasets are primarily based on Wikipedia articles. Wikipedia articles are available under the Creative Commons Attribution-ShareAlike 4.0 International License, allowing us to remix, transform, and redistribute the material.

In the following, we describe the data generation and format in more detail and perform a bias analysis as a sanity check to verify that the dataset is unbiased.

1.2 Data Generation

We accessed the top 100 list of popular books on Project Gutenberg on March 17, 2022, and to obtain the corresponding Wikipedia articles, we ran Google queries. For web scraping, we use the software Selenium[1]. After scraping the sentences from the Wikipedia articles of the books, we preprocess the sentences using the Python library Spacy[2].

Below, we provide additional details of our data processing rules as part of the data generation process. We employ Spacy to only include sentences with root verbs in the 3rd person singular.

The objective of this dataset is to construct a ground truth resource for evaluating language models under controlled grammatical-gender manipulations. To this end, we created a set of manipulated sentences, each available in three variants: male, female, and non-binary. Depending on the manipulation scheme, sentences either (i) replace only the subject's gendered words or (ii) replace all gender-related words (subjects, objects, and modifiers) to produce fully male or fully female versions. The inclusion of non-binary variants was intended to provide a more inclusive basis for bias research, though we acknowledge that the representation of gender through pronoun alternation remains a simplification.

1.2.0.1 Sentence segmentation and filtering

We segmented over 5000 paragraphs into candidate sentences using the small English model of Spacy, yielding more than 19,500 sentences. Spacy's syntactic annotations were then leveraged to filter the sentences with: (i) root verb selection: we kept only sentences whose root verb was in third-person singular, determined from Spacy's morph feature annotations. (ii) subject extraction: subjects were identified using the dependency relation nsubj (or closely related subject arcs) relative to the root verb. We converted Spacy's dependency generator to a list for each candidate sentence. (iii) sentence length constraint: to improve clarity and reduce annotation burden, we discarded sentences with more than 30 tokens. (iv) punctuation filter: sentences not ending with a period were removed. (v) exclusion of "it" subjects: we filtered out sentences where the subject was "it", as these do not contribute to gender-based evaluation.

Identification of human-related subjects To restrict the dataset to human-relevant entities, we retained only subjects categorized as: (i) proper nouns (Spacy: token.pos_ == "PROPN") referring to characters.

[1] https://www.selenium.dev/

[2] https://spacy.io

Author names and spurious references (e.g., scholars writing about the book) were manually removed. (ii) pronouns ("he"/"she"), detected by Spacy's morph features: PronType=Prs and Gender $\in \{\text{Masc}, \text{Fem}\}$. (iii) common nouns (e.g., father, sister, boy, girl), which were manually screened to ensure they referred to human roles rather than misclassifications or irrelevant objects. To identify human-related nouns, we compiled lists for each group (proper nouns, pronouns, common nouns) and manually validated them. Single-occurrence names were typically excluded, as they often referred to non-plot entities.

1.2.0.2 De-duplication and manual review

We removed duplicate sentences and further manually inspected candidate sentences with a custom visualization tool, highlighting the detected subject and root verb. Sentences that were malformed, fragmentary, or unrelated to the plot (e.g., citations, chapter headings, or errors in Spacy segmentation) were excluded.

1.2.0.3 Sentence annotation and gender manipulation

From this filtering process, we obtained 4,830 sentences. Each was then manually annotated using a custom-built web interface[3]. Annotation was conducted by four annotators, with disagreements resolved through discussion. Inter-annotator agreement was not formally measured (given the deterministic nature of replacements), but the four annotators reviewed every sentence, and residual errors were corrected.

For each sentence, we generated three variants: (i) male version: subjects, pronouns, and gendered words were replaced with male forms (e.g., she $\rightarrow$ he, daughter $\rightarrow$ son). (ii) female version: parallel substitutions for female forms. (iii) non-binary version: we used the singular they pronoun, adjusting possessives (her $\rightarrow$ their) and verbs for agreement (she thinks $\rightarrow$ they think). Proper names were replaced with the appropriate pronoun (e.g., Paul did $\rightarrow$ He did, She did, They did). Gender-indicative common nouns (e.g., son, brother, king) were mapped to gendered counterparts, with neutral forms chosen where available (sibling, monarch, child). Special care was taken to ensure that verb agreement remained grammatical in the non-binary variants.

This pipeline resulted in two ground-truth datasets: $\mathcal{D}_S$: only subject terms are manipulated. $\mathcal{D}_A$: all gendered terms are manipulated.

Together, these datasets provide 3 variants of 1,610 base sentences (9,660 total), each aligned at the token level and accompanied by word-level labels for ground-truth feature attributions.

We attempted to employ fully automated sentence labeling using GPT-4 OpenAI (2023), but encountered inconsistencies in identifying names, genders, and gendered terms, as well as detecting human subjects, particularly in dataset $\mathcal{D}_S$. Due to the need for precise ground truth labels to benchmark various explanation methods, we opted for a manual labeling approach instead.

1.3 Data Format

The datasets are available in the following folder structure.

```
GECO
├── data_config.json
└── gender_all
    └── test.jsonl (644 Sentences)
```

[3] https://github.com/braindatalab/gecobench/tree/main/data/dataset_generation

Table S1. Labeling rules for the two dataset variants. $\mathcal{D}_S$ manipulates only the subject term, while $\mathcal{D}_A$ manipulates all gender-related words.

$\mathcal{D}_S$: **Subject-only manipulations**	$\mathcal{D}_A$: **All gender-related manipulations**
• Replace only the grammatical subject (proper nouns, pronouns, or human-related common nouns).<br>• Proper names → pronouns (e.g., *Paul did → He/She/They did*).<br>• Pronoun subjects: *he/she/they*.<br>• Subject common nouns: *son → daughter → child.*<br>• Verb agreement corrected for non-binary forms (*She thinks → They think*).<br>• All other tokens remain unchanged.	• Replace all gendered tokens: subjects, objects, modifiers, kinship terms.<br>• Apply subject rules as in $\mathcal{D}_S$.<br>• Objects: *her brother → his sister → their sibling*.<br>• Possessives: *his/her/their.*<br>• Kinship/titles: *king → queen → monarch, father → mother → parent.*<br>• Ensure correct verb agreement with singular *they*.<br>• Sentences systematically checked for grammatical well-formedness, otherwise removed.

```
│
│   └─train.jsonl (2576 Sentences)
└─gender_subj
    ├─test.jsonl (644 Sentences)
    └─train.jsonl (2576 Sentences)
```

The sentences are available in JSONL files, with each line representing a sentence in either male or female form. An example sentence can be seen in Listing 1. Each line contains the input sentence as a list of words, as well as the explanation ground truth for each word. The "target" field indicates whether the sentence is in female form (0) or male form (1). Lastly, the "sentence_idx" field identifies which original sentence was altered and can be used to match the male and female forms of a sentence.

Listing 1. Example sentence of the class $\mathbf{x}_A^M$.

```
{
  "sentence": ["Paul", "loves", "his", "dog"],
  "ground_truth": [1.0, 0.0, 1.0, 0.0],
  "target": 1,
  "sentence_idx": 0
}
```

2 MODELS

In the following, we provide further details about the model training and performance. All models are implemented in PyTorch.

2.1 Training

For model training, we split the two datasets $\mathcal{D}_S$ and $\mathcal{D}_A$ into training and test sets $\mathcal{D}_S^{train}$, $\mathcal{D}_S^{test}$ and $\mathcal{D}_A^{train}$, $\mathcal{D}_A^{test}$. We train 5 repetitions for each model, using a different random seed for each repetition. This

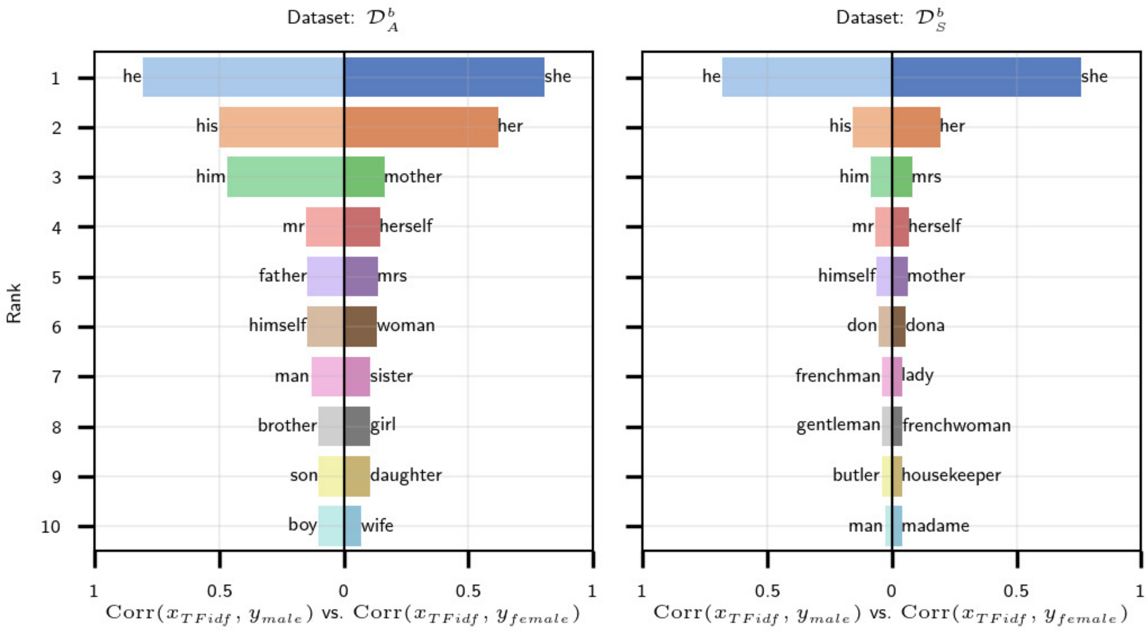

Figure S1. Pearson correlation between tf-idf representation of words and the target for GECO. Here, we see the top ten words by correlation, and labeled words such as the pronouns *he* and *she* or *his* and *her* are consistently ranked highest in both datasets $\mathcal{D}_A$ and $\mathcal{D}_S$, indicating how the labeling introduced dependency between target and words.

is done not only to compensate for variations in model performance Dodge et al. (2020); Bugliarello et al. (2021) but also to capture the resulting variance in model explanations.

We optimize the learning rate and keep the remaining hyperparameters fixed. Table S2 shows a full overview of all hyperparameters and values we use. While it could be interesting to explore how various training parameters impact explanation performance, our focus is on achieving the same accuracy threshold across all models. Therefore, we do not use the same hyperparameters for all models. All models were trained on our internal cluster with an Nvidia A100 (40GB) GPU. For hyperparameter optimization and the final model training across the five different training schemes and two datasets, we conducted 464 training runs with an average running time of 68 seconds.

An overview of the performance of the models on the training, validation, and test sets is shown in Figure S2.

**Models**	Batch Size	Embedding Dimension	Epochs	Learning Rate
BERT-C	32	768	20	0.01
BERT-CE	32	768	20	0.0001
BERT-CEf	32	768	20	0.01
BERT-CEfAf	32	768	20	0.000005
OLA-CEA	64	64	200	0.01

Table S2. Overview of BERT training schemes and values of the hyperparameters used to train them.

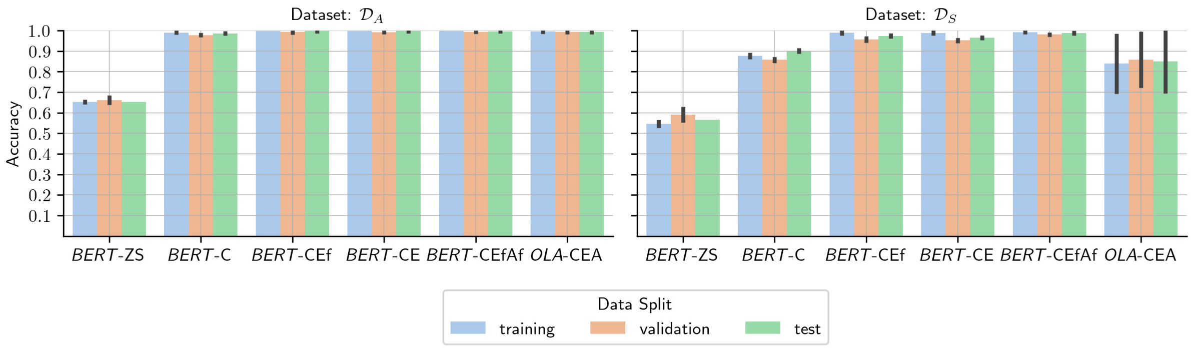

Figure S2. Model performance.

3 EXPLANATIONS

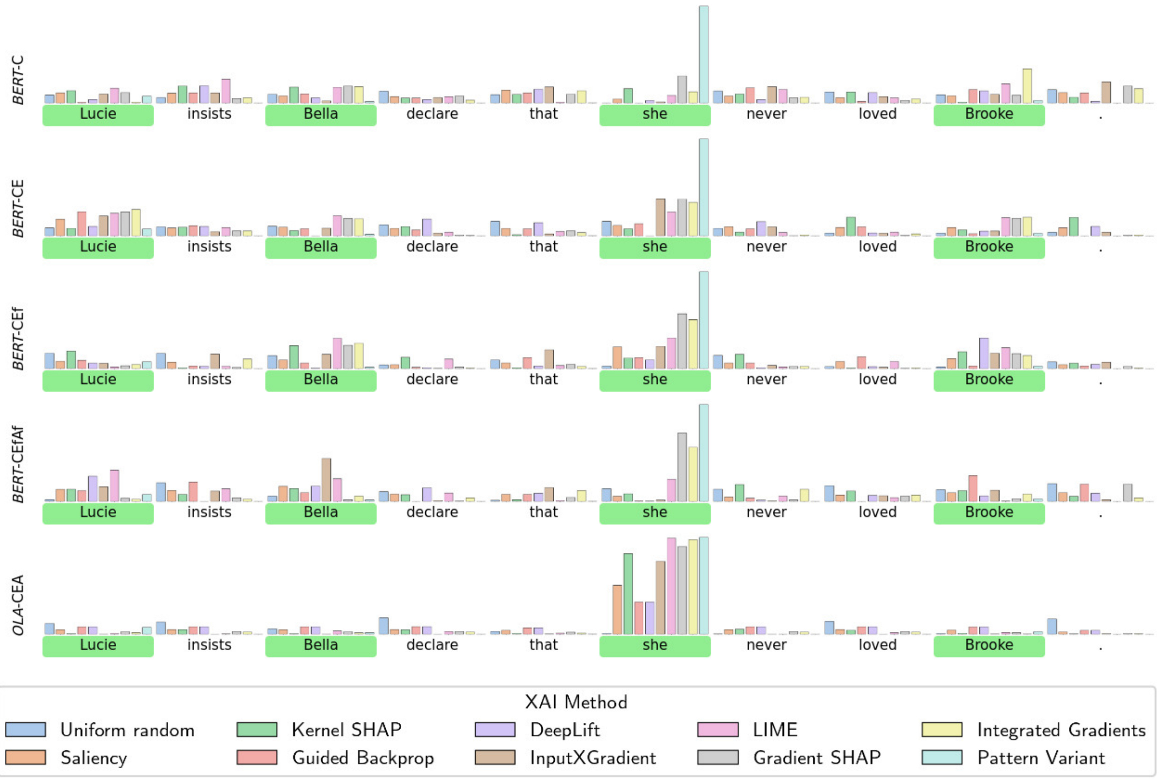

Figure S3. Feature attributions by popular explanation methods for one sample sentence, broken down into input tokens as given to the respective model, with the ground truth manipulations highlighted in green. The majority of importance by many methods is correctly attributed to the word "she"; however, all tokenized words show non-zero attribution for multiple methods, including the character period ".".

REFERENCES

Bugliarello, E., Cotterell, R., Okazaki, N., and Elliott, D. (2021). Multimodal Pretraining Unmasked: A Meta-Analysis and a Unified Framework of Vision-and-Language BERTs. *Transactions of the Association for Computational Linguistics* 9, 978–994. Place: Cambridge, MA Publisher: MIT Press

Dodge, J., Ilharco, G., Schwartz, R., Farhadi, A., Hajishirzi, H., and Smith, N. (2020). Fine-tuning pretrained language models: Weight initializations, data orders, and early stopping. *arXiv preprint arXiv:2002.06305*

OpenAI (2023). GPT-4 technical report. *CoRR* abs/2303.08774. doi:10.48550/ARXIV.2303.08774
